# The Changing Epidemiology of Human African Trypanosomiasis among Patients from Nonendemic Countries –1902–2012

**DOI:** 10.1371/journal.pone.0088647

**Published:** 2014-02-19

**Authors:** Ami Neuberger, Eyal Meltzer, Eyal Leshem, Yaakov Dickstein, Shmuel Stienlauf, Eli Schwartz

**Affiliations:** 1 Unit of Infectious Diseases and Internal Medicine B, Rambam Medical Center and Bruce Rappaport Faculty of Medicine, Technion Institute of Technology, Haifa, Israel; 2 The Center for Geographic Medicine and Internal Medicine A and C, Sheba Medical Center and Sackler Faculty of Medicine, Tel Aviv University, Tel Aviv, Israel; 3 Internal Medicine A, Rambam Medical Center, Haifa, Israel; Instituto de Higiene e Medicina Tropical, Portugal

## Abstract

**Background:**

Although human African trypanosomiasis (HAT) is uncommon among patients from non-endemic countries (NEC), there has been an increase in the number of cases reported in recent years.

**Methods:**

A systematic review of the literature was performed. The number of incoming tourists to HAT endemic countries was obtained from the United Nations World Tourism Organization. All HAT cases diagnosed in patients from NEC were included. Immigrants and refugees were excluded. We compared patients during and after the colonial period, and analyzed the relationship between the number of incoming travellers and the number of HAT cases.

**Results:**

Between 1902 and 2012, HAT was reported in 244 patients. Most HAT cases were reported before 1920, and after the year 2000. In the colonial era the average age of patients was lower (32.5±7.8 vs. 43.0±16.1 years, P<0.001), the proportion of females was lower (10.0% vs. 23.9%, P<0.01], most cases were diagnosed in expatriates, missionaries and soldiers (74.3%), and Gambian trypanosomiasis accounted for 86/110, (78%) of cases. In the post-colonial era most patients 91/125 (72.8%) were short-term tourists to game parks in Eastern and South-Eastern Africa (mainly in Tanzania); Rhodesian trypanosomiasis accounted for 94/123 (76.4%) of cases. Between 1995 and 2010 there has been a constant linear increase in the number of incoming tourists to Tanzania, and HAT cases occurred in small outbreaks rather than following a similar linear pattern.

**Conclusions:**

In recent decades HAT patients from NEC are older, and more likely to be tourists who acquired the disease while visiting game-parks in Eastern and South-Eastern Africa. While Rhodesian trypanosomiasis is relatively uncommon among Africans, it now accounts for most cases reported among patients from NEC. Returning febrile travellers without an alternative diagnosis should be evaluated for HAT. Cases among travellers may serve as sentinels for Rhodesian trypanosomiasis “hot spots” in Africa.

## Introduction

John Atkins, an eighteenth-century English naval surgeon, observed multiple cases of what he called a “sleepy distemper”. Atkins assumed that the disease afflicted only Africans and was related to a “natural weakness of the brain” [1]. However, cases of Human African Trypanosomiasis (HAT) were eventually reported among non-Africans, and trypanosomes were actually observed for the first time in the blood of an English steamboat captain who had contracted HAT in Gambia in 1902 [2]. Eight years later *Trypanosoma rhodesiense* was observed for the first time in another English patient [3]. In the 110 years that have elapsed since, there have been multiple reports of HAT among patients from nonendemic countries (NEC) [4]–[5].

HAT is caused by the protozoan parasite *Trypanosoma brucei*, and transmitted by the bite of the tsetse fly of genus *Glossina* [6]. Rhodesian trypanosomiasis is caused by *Trypanosoma brucei rhodesiense*, which commonly infects wild and domestic animals in the savannahs and woodlands of East and South-East Africa. Incidental infection of humans usually implies close contact with the tsetse fly, which feeds on the main animal reservoirs, namely antelopes and cattle. People living or working in close vicinity to animals are therefore at an increased risk of contracting the disease – as are travelers who may be infected in the nature reserves of Eastern and South-Eastern Africa.

The etiologic agent of Gambian trypanosomiasis is *Trypanosoma brucei gambiense*, which is endemic in the humid, forested areas of Central and West Africa. Infection with the Gambian form of trypanosomiasis accounts for more than 95% of HAT cases in Africa, and is transmitted by tsetse species that preferentially feed on humans. Animals are hence not important reservoirs of West African trypanosomiasis, and the transmission usually occurs in rural areas where vectors and humans come into close contact around water holes. Both forms of HAT, if left untreated, ultimately lead to death.

Over the past 110 years the epidemiology of trypanosomiasis in Africa evolved markedly [6]–[7]. In recent years, enhanced surveillance and control efforts resulted in a steady decline in the number of new cases of HAT among Africans [8]. However, sporadic cases and small outbreaks of HAT are reported increasingly in patients from NEC [5]. In addition, there has also been a change in the number of visitors from NEC, the purpose of their visit, their length of stay, and in the diagnostic and therapeutic interventions used in the care of patients with HAT. In the light of these changes, we assumed that the demographic “profile”, travel history, and clinical manifestations of patients with HAT coming from non-endemic countries have changed considerably since 1902.

## Methods

A systematic review of the literature was performed. All cases of HAT reported in the medical literature in all languages since 1902 in patients from NEC were included. These patients included both short-term visitors and expatriates who were born in a NEC but were living in Africa when infected. Cases were identified through the PubMed and ProMED databases; we used the following search strategy: ((sleeping sickness OR human African trypanosomiasis) AND (epidemiology OR travel)). References from relevant articles were reviewed as well. Cases that were reported from the same country in the same year, and included patients of matching age and gender, were presumed to be identical and reported only once. Cases of HAT among immigrants and refugees from Africa were excluded, as were cases in which the country of origin was not explicitly specified. For cases reported before 1967 we used the review by Duggan et al. as we located only one pre-1967 HAT case that had not been included in Duggan’s article [4]. We recorded each patient’s demographic and clinical data.

Patients were determined to have East or West African trypanosomiasis according to microbiologic results or to their travel history. If a patient moved between several different countries in which only one subspecies of *Trypanosoma brucei* is endemic we designated only East or West Africa as the likely place of infection. We could not determine the subspecies involved and the country in which the infection was acquired, if microbiologic data did not include subspecies identification, and if travel history was either not reported or included Uganda which is endemic for both types of *Trypanosoma brucei* subspecies.

A comparison of demographic and epidemiologic data between patients during and after the colonial period was performed. Data regarding the number of visitors to endemic countries were obtained with permission from the United Nations World Tourism Organization (UNWTO). Only visitors from NEC were included, and the change in the annual number of incoming tourists to HAT–endemic countries was compared to the change in number of cases reported in such countries.

Comparison of patients’ characteristics patients diagnosed with HAT during and after the colonial period was performed by using chi-square test. Age was compared using the Student’s T-test. Two-tailed *p* values of 0.05 or less were considered as statistically significant. The risk for disease per 100,000 visitors was calculated by using reported cases as numerator, and the estimated total number of incoming travelers from the UNTWO database as denominator. All statistical analyses were performed with using SPSS (Statistics Products Solutions Services) 18.0 software for Windows.

For comparison of the colonial and the postcolonial periods, we have used 1966 as a cut-off, as nearly all countries with reported cases of HAT among patients from NEC had gained independence by then. Exceptions include Equatorial Guinea (1968), Angola (1975), Mozambique (1975), and Zimbabwe (1980).

Some geographic entities that no longer exist appear in the pre-1960s literature. Despite certain differences between these two territories, we assumed The Cameroons to be included in modern-day Cameroon (Northern Cameroons were actually incorporated into Nigeria). Similarly Tanganyika is now a part of modern Tanzania, Belgian Congo is the Democratic Republic of Congo, and Nyasaland is Malawi. The British colony of Southern Rhodesia and Rhodesia itself are modern day Zimbabwe, whereas Northern Rhodesia is now Zambia. Fernando Pó is the island Bioko which is now part of Equatorial Guinea, Bechuanaland is Botswana, and the term “Gold Coast” refers to modern day Ghana.

## Results

**Figure 1:**
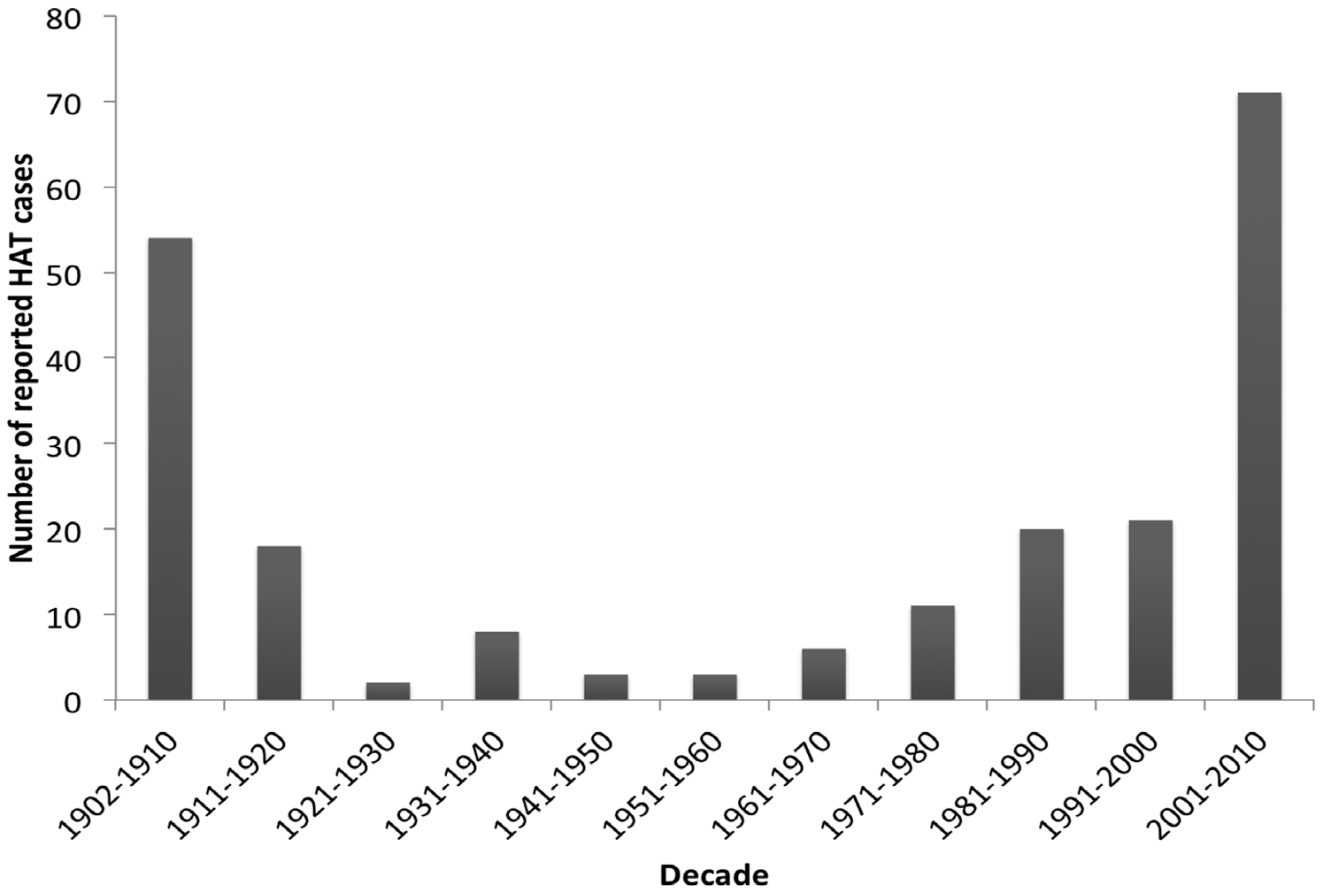
Number of reported HAT cases among patients from non-endemic countries per decade.

The search yielded 1211 articles of which 1170 were deemed irrelevant and 41 articles that included 303 cases of HAT diagnosed outside of Africa or occurring among non-Africans. We excluded 38 cases of HAT diagnosed among African expatriates or African refugees, and 21 additional cases reported more than once. Between the years 1902 and 2012, the number of HAT cases reported in patients from NEC totaled 244 [2]–[3], [5], [9]–[34]. The number of HAT cases per decade is shown in Figure 1. A “J” shaped curve with relatively more cases of HAT reported in the first two decades of the twentieth century (an average of 36 per decade), and then between 1991 and 2000 (21 cases per decade) and between 2001 and 2010 (71 cases per decade) can be seen. Only 24 cases were reported in the fifty years that elapsed between 1921 and 1970.

The average age of all patients was 38.5±14 years (range 1–74). In the colonial era the average age was lower than the age of patients diagnosed in the postcolonial era (table 1). Overall, most patients (82.8%) were male. However, in the colonial period 10% were female, whereas this proportion increased to 23.9% in the postcolonial period (Table 1). The purpose of visit to Africa was reported in 86/110 and 125/134 of the patients diagnosed in the colonial and postcolonial eras, respectively. Until the mid-1960s 74.3% of HAT cases were diagnosed in expatriates, missionaries, and soldiers. However, after 1966 most patients (72.8%) were tourists, with a smaller proportion of cases found among missionaries, soldiers, and expatriates (Table 1).

**Table 1:** Epidemiologic data and mortality rates of patients from non-endemic countries who acquired Human African Trypanosomiasis during the colonial period (1902–1966) and during the post-colonial period (1967–2012).

	Colonial period	Post-colonial period	P-value
Age, years (average±SD)	32.5±7.8	43.0±16.1	p<0.001
Gender, females/all patients (% female)	11/110 (10%)	32/134 (23.9%)	p<0.001
Occupation/purpose of visit:			p<0.001
• Tourist	0/86 (0%)	91/125(72.8%)	
• Expatriate	34/86 (39.5)%	14/125 (11.2%)	
• Missionary	15/86 (17.4%)	3/125 (2.4%)	
• Military	15/86 (17.4%)	4/125 (3.2%)	
• Others*	22/86 (25.6%)	13/125 (10.4%)	
*T. brucei* subspecies**:			p<0.001
• T. b. rhodesiense (%)	24/110 (21.8%)	98/128 (76.6%)	
• T.b. gambiense (%)	86/110 (76.2%)	30/128 (23.6%)	
Mortality (%)	6/43(14%)Ŧ	4/98(4.1%) E HAT 1/30 (3.3%) W HAT	N/A

SD, standard deviation; E HAT, East African Trypanosomiasis; W HAT, Western African Trypanosomiasis;

*Others : Medical personnel, teachers, sailors, scientists;

**Subspecies according to microbiologic diagnosis or likely place of infection;

Ŧ mostly West African Trypanosomiasis.

N/A – not applicable.

**Figure 2:**
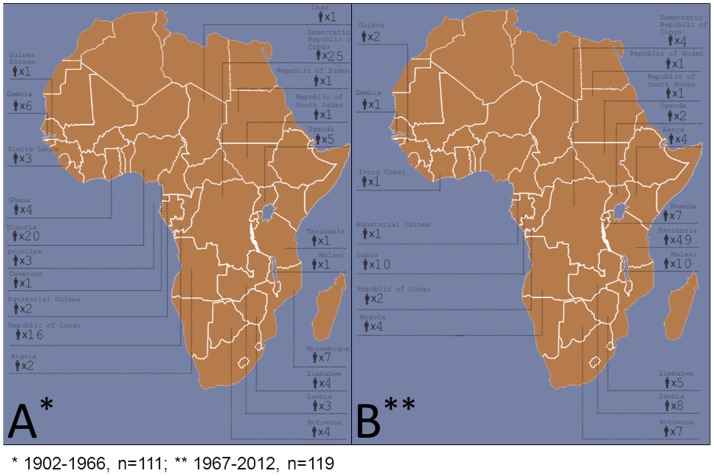
Geographical distribution of HAT cases among patients from non-endemic countries in the colonial (1902–1966) (A) and post-colonial (1967–2012) (B) periods.

The geographic distribution of HAT acquisition before and after 1966 was also different. Most cases of HAT described in the colonial period (86/110, 78%) were acquired in Western African countries where only *T. b. gambiense* is endemic. During the postcolonial period the vast majority of cases were identified among tourists visiting game parks in East Africa, most commonly in Tanzania (Figure 2). This trend is reflected by a shift in the dominant *Trypanosoma brucei* subspecies. *Trypanosoma brucei rhodesiense* accounted for 98/128 (76.6%) of all cases with subspecies identification that occurred after 1966. (Table 1).

**Figure 3:**
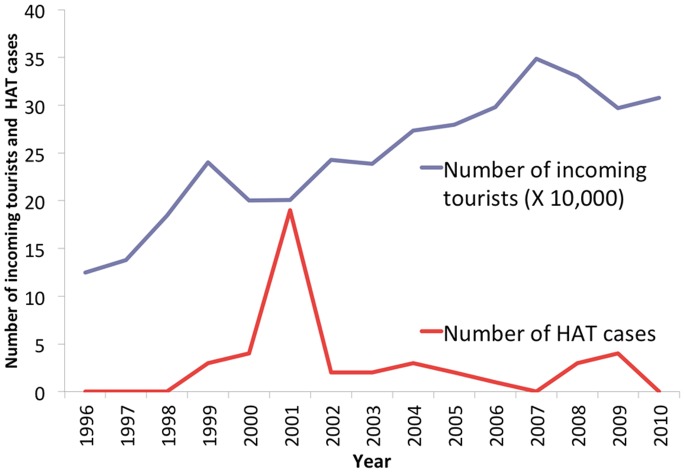
Number of incoming tourists and number of HAT cases among patients from non-endemic countries in Tanzania during 1995–2010.

After 1990 tourists that were infected in Tanzania accounted for 47/100 (47%) of all cases. According to the data provided by the UNWTO, between 1995 and 2010, the number of travelers entering Tanzania increased steadily. However, the change in the number of reported cases per year did not correspond to the increase in the number of incoming tourists. Rather, single cases continued to occur sporadically, interspersed with small outbreaks (Figure 3). The yearly risk for acquiring HAT in Tanzania varied greatly between 1995 and 2010 (0–9.49/100000 visitors), but was ≤2 in all years except 2001. The same pattern was also observed in Malawi and Zambia where repeated cases of HAT have been reported among tourists in recent years (data not shown). The number of cases in other African countries was too small for such an analysis to be performed.

In the vast majority of Rhodesian trypanosomiasis cases, diagnosis was made by demonstrating trypanosomes in a peripheral blood smear [21/24 (87.5%) during the colonial period, and 94/98 (95.9%) during the post-colonial period]. Peripheral blood smear yielded a diagnosis in 60/86 (69.8%) of patients with Gambian trypanosomiasis during the colonial period, and 24/30 (80%) during the post-colonial era (p<0.01 for the comparison of the proportion of cases of East and West African trypanosomiasis diagnosed by peripheral blood smear). Lymph node aspiration and CSF analysis yielded a diagnosis in a higher proportion of patients with Gambian trypanosomiasis (Table 2). In 11 cases the diagnostic test used was not described, and in three cases (all from the older literature) diagnosis was made on clinical grounds alone.

**Table 2:** Diagnostic means of Human African Trypanosomiasis among patients from non-endemic countries who acquired Human African Trypanosomiasis during the colonial period (1902–1966) and during the post-colonial period (1967–2012)#.

Colonial period	West African Trypanosomiasis (N = 86)	East African Trypanosomiasis (N = 24)
Microscopy – blood	60	21
Microscopy – lymph node	18	1
Microscopy – CSFŦ	7	2
Post Mortem	7	0
Clinical Diagnosis only/not reported	3	0
**Post colonial period** #	**West African Trypanosomiasis (N = 30)** ŧ	**East African Trypanosomiasis (N = 98)** ŧ
Microscopy – blood	24	94
Microscopy – lymph node	5	0
Microscopy – CSFŦ	6	7
Polymerase chain reaction	1	2
Bone marrow biopsy	5	3
Chancre biopsy and microscopy	0	3
Rat innoculation	0	1
CATT±	1	0
immunofluorescence	0	2
Not reported	4	7

# Several cases were confirmed in more than one type of specimen or by more than one laboratory technique;

Ŧ CSF, cerebrospinal fluid;

ŧ In six HAT cases the subspecies type could not be determined;

± CATT. card-agglutination test for trypanosomiasis.

In the colonial period, 6/43 (14%) of European patients, who were diagnosed after the antitrypanosome medications had become available, succumbed to HAT. Precise data is not available, but nearly all of these patients had Gambian trypanosomiasis. Of the patients diagnosed in the post-colonial period, and whose outcome and subspecies identification were available, 4/98 (4.1%) and 1/30 (3.3%) of patients with Rhodesian and Gambian African trypanosomiasis, respectively, died of the disease (Table 1). All patients diagnosed before 1912 either died or were lost to follow up.

## Discussion

The epidemiology of HAT among patients from NEC has changed between 1902 and 2012. The number of reported cases per decade varied greatly, with a large increase after the year 2000. In addition, patients in recent decades are somewhat older, are more likely to be tourists, and to have acquired the disease while visiting game parks in Eastern and South-Eastern Africa. *T. B. rhodesiense* is now implicated in the vast majority of HAT cases among patients from NEC, whereas Gambian trypanosomiasis was much more common during the colonial era. The mortality rate of HAT among patients from NEC has probably decreased in recent years.

Since the “opening up” of Africa, the often-complex interaction between Africa and the outer world have been closely related to the history of HAT. The early colonial era coincided with civil wars, population displacement, and forced transfer of slaves afflicted with HAT [35]–[36]. In some areas (e.g. Belgian Congo) the use of forced labour contributed to the spread of the disease among the poor and the displaced [35]. At the beginning of the twentieth century, trypanosomes were identified as the agents of African sleeping sickness, and the role of the tsetse fly in its transmission was finally elucidated [35]. Arsenicals were introduced in 1912, and suramin became widely available only after 1916. Thus, in the beginning of the twentieth century, social conditions and the lack of both effective treatment and efficient vector control favoured the transmission of HAT among local population as well as visitors.

The decrease in the number of reported HAT cases among patients from NEC in the mid-twentieth century most likely reflects the gradual decrease in overall HAT incidence in Africa. Systemic case detection, mass treatment programs, increased availability of treatment, and vector control resulted in a dramatic decrease in HAT incidence before the 1960s. Transmission was interrupted in most areas, and elimination of HAT as a public health problem seemed feasible [8].

Since the 1960s most HAT cases were reported in the Democratic Republic of Congo, Angola, Uganda, Sudan, Central African Republic and Chad where reemergence of HAT has often been linked to civil wars, political unrest, and scarcity of resources [6], [8], [37]–[40]. In addition, HAT was no longer seen as a major healthcare problem, and control programs often became more relaxed. As a result HAT reemerged in many countries, and its incidence continued to rise until the late 1990s [35].

Renewed efforts by national healthcare systems, nongovernmental organizations and the WHO, lead to a 63% decrease in the number of newly reported cases between 2000 and 2009 [7], [8]. Paradoxically, our data show that the number of cases reported among patients from NEC increased markedly after the year 2000, whereas the number of cases of both forms of HAT among Africans decreased rapidly in the same period of time [8]. This apparent discrepancy is probably explained by changes in HAT epidemiology that occurred among patients from NEC during the past several decades, and by more complete reporting of cases in recent years.

Themissionaries, soldiers, and colonial administrators who resided in rural Africa at the turn of the twentieth century were largely replaced by short-term tourists, who often include the big nature reserves of East Africa in their itinerary. In 1958, for example, out of 109,435 Europeans living in Belgian Congo, 57.6% were “technicians”, 8.6% civil servants, and 6.5% missionaries. Most of these people were living in rural areas, and at risk of Gambian HAT [41]. In sharp contrast, in recent years the vast majority of people from NEC in Africa are short-time tourists to East Africa and the countries with the highest HAT incidence are hardly ever visited by today’s tourists. As a result of these changes, the number of cases of Gambian trypanosomiasis among patients from NEC has decreased, and there has been a sharp increase in the number of Rhodesian trypanosomiasis cases diagnosed among tourists.

About half of the cases of HAT diagnosed in patients from NEC in recent decades occurred in Tanzania. The risk of a traveler to acquire HAT in Tanzania is small, but fluctuates widely, and does not correlate directly with the number of incoming tourists to the country (figure 3). Similar findings were observed in other countries like Malawi and Zambia that have a growing tourist industry. The lack of correlation between the number of HAT cases and the number of incoming tourists probably results from the fact that East African trypanosomiasis tends to occur in relatively small “pockets” or “hot spots” of endemicity [42]. As such, single cases and small outbreaks are the rule, and widespread epidemics are nowadays a rarity in most African countries. A recent study identified several high-risk activities among travelers who acquired Rhodesian trypanosomiasis; namely, visiting game parks (77.2%), hunting (8.8%), and participation in military activity (5.2%) [43]. Local changes in tourist travel patterns (e.g. different itineraries, closer contact with wildlife that serve as tsetse reservoirs, and longer stays in tsetse fly-infested areas), are probably also related to the risk of acquiring HAT.

The geographic distribution of HAT cases among returning travelers may contribute to disease surveillance in Africa, as travelers occasionally play a role as “sentinels” for detecting disease “hot-spots” in low-resource countries [7], [44]. A recent outbreak of Rhodesian trypanosomiasis among tourists visiting the Serengeti national park in Tanzania during 2001, for example, provided important data leading to the implementation of preventive measures, and a subsequent decline in the number of tsetse flies in that area [45]. To the best of our knowledge, hotspots of Gambian HAT have not been identified through the diagnosis of expatriates or tourists.

The lower mortality rate among patients during the post-colonial era is hard to interpret in light of the small number of fatal cases (Table 1). Possible explanations include better accessibility to healthcare, and higher rates of Rhodesian trypanosomiasis which is more easily diagnosed.

There are several important limitations to our study. The vastly different ways data were collected and became available over a 100 year span limit the accuracy of our findings. The number of reported HAT cases is probably an underestimate. Some cases were probably never diagnosed correctly in developed countries, where doctors are familiar neither with the clinical manifestations of HAT, nor with the limitations of the various diagnostic modalities available today [46]. Death in such cases was probably erroneously attributed to other causes. In addition, before the advent of Internet-based reporting systems like the GeoSentinel, ProMED mail, and TropNetEurop in the 1990s, some cases of HAT probably went unreported. Data before the year 2000 relied on case reports and case series, while Simarro et al. based their report on WHO supply of drugs in NEC [5]. Since antitrypanosome medications in NEC are distributed solely by the WHO, this report essentially includes all patients treated in such countries (with the uncommon exception of patients treated only with pentamidine). This difference in the source of data explains the fact that Migchelsen et al., in a report based on a literature search of all cases occurring between 1990 and 2010, have detected 32 cases between 2000 and 2010, whereas Simarro et al have reported 74 HAT cases among tourists for the same period [5], [9]. Therefore, the large increase in the number of HAT cases among tourists after the year 2000 is to be explained, at least partially, by more complete reporting. The designation of “East” and “West” African trypanosomiasis may be inaccurate, since many of the “colonial” were categorized as East or West African trypanosomiasis based on travel history only. Both diseases exist concurrently in some areas of Uganda, and travelers’ itineraries obtained through retrospective interviews may be inaccurate [47]–[48]. Moreover, microbiological reports are not always available or complete. Since nearly all “colonial” cases were acquired in Europeans living for prolonged periods in West Africa, and since most “post-colonial” cases were acquired by short-term tourists visiting only East African game parks, we are certain that the designation of “East” or “West” African trypanosomiasis in the cited cases is accurate.

The different clinical course of Rhodesian and Gambian trypanosomiasis may have led to under diagnosis of the latter. Gambian trypanosomiasis is characterized by two distinct stages. The first (haemolymphatic) stage is typified by fever and lymphadenopathy; the “classic” neurologic manifestations may occur years later, during the second (meningoencephalitic) stage of the disease. On the other hand, Rhodesian trypanosomiasis is a more acute disease. Rhodesian trypanosomiasis may be easier to diagnose among travelers as a recent trip to Africa is more likely to be reported, both fever and a chancre at the initial tsetse fly bite site are common, and neurologic symptoms tend to appear earlier in the course of the disease. In conclusion, there has been a change in the epidemiology of the disease among patients from NEC over the past century, and there has been an increase in the number of reported cases over the past two decades. In the colonial era patients were likely to be male Europeans in their thirties who had come to West or Central Africa as missionaries, soldiers, and settlers, and were afflicted with Gambian trypanosomiasis. In the postcolonial era, patients from NEC are somewhat older, are likely to be short-term tourists visiting the great nature reserves of East and South East Africa, and commonly present with an acute febrile illness typical of Rhodesian trypanosomiasis. Elimination of HAT as a public health problem has now become a realistic goal in most African countries. Until that day, all returning travelers with a compatible febrile illness, and without an alternative diagnosis, will have to be evaluated for HAT. A timely diagnosis will not only ensure better care of the patient, but also aid in detecting Rhodesian trypanosomiasis “hot spots” in Africa [44].

## Supporting Information

Checklist S1 PRISMA checklist.
(DOC)

Flow Diagram S1 PRISMA Flow Diagram.
(DOC)
