# Supplementary material for: The Changing Epidemiology of Human African Trypanosomiasis among Patients from Nonendemic Countries –1902–2012
Source: PLoS One. 2014 Feb 19;9(2):e88647. doi: 10.1371/journal.pone.0088647 (PMC3929605; doi:10.1371/journal.pone.0088647)
Supplement: Flow Diagram S1 — PRISMA Flow Diagram. (DOC) [file pone.0088647.s002.doc]

**Human African Trypanosomiasis Patients from Non-endemic Countries – Data extraction (according the Prisma – transparent reporting of systematic reviews and meta-analyses- 2009 flow diagram)**

**Screening**

**Included**

**Eligibility**

**Identification**

Records identified through database searching
(n =1202 )

Additional records identified through other sources
(n = 9 )

Records after duplicates removed
(n =1211 )

Records screened
(n = 1211 )

Records excluded
(n = 1170 )

Full-text articles assessed for eligibility
(n =41, cases=303 )

Cases excluded (n =59, 38 cases occurring among refugees and 21 cases reported more than once).

Cases included in qualitative synthesis
(n = 244 )

Cases included in quantitative synthesis
(n = 244 )
